# River transport of cattle in northern Brazil - a systematic review

**DOI:** 10.1007/s11250-026-05054-1

**Published:** 2026-06-01

**Authors:** Welligton Conceição da Silva, Éder Bruno Rebelo da Silva, Italo Messias Ferreira de Souza, Vanessa Sousa Pinto, Luís Gustavo Paixão Vilela, Carlos Eduardo Lima Sousa, Thiago Nogueira da Silva, Alinne da Silva Souza, João Paulo de Sousa Cunha, Vinicius Costa Gomes de Castro, Raimundo Nonato Colares Camargo-Júnior, Rubens Lima de Andrade, Jucelane Salvino de Lima, Lílian Kátia Ximenes Silva, André Guimarães Maciel e Silva, José de Brito Lourenço Júnior, Jamile Andréa Rodrigues da Silva, Marina de Nadai Bonin Gomes, Cláudio Vieira de Araújo

**Affiliations:** 1 Federal University of Mato Grosso, Sinop, Brazil; 2 Lutheran University Center of Santarém (Ceuls), Santarém, Pará Brazil; 3 University Center of the Amazon (UNAMA), Santarém, Pará Brazil; 4 Federal Rural University of the Amazon, Santarém, Pará Brazil; 5 Federal Institute of Pará (IFPA), Santarém, Pará Brazil; 6 Federal University of Western Pará (UFOPA), Santarém, Pará Brazil; 7 Federal University of Pará (UFPA), Castanhal, Pará Brazil; 8 Federal University of Mato Grosso Do Sul (UFMS), Santarém, Pará Brazil

**Keywords:** Heat stress, Vessels, Amazon livestock, Welfare

## Abstract

The river transport of cattle by barges is a common practice in western Pará, where rivers are the main access routes to livestock production, slaughter, and marketing areas. In this context, the long journeys made by boats or ferries adapted for cattle transport can expose animals to conditions of thermal stress, due to the high relative humidity of the region, temperature and humidity fluctuations, and wind speed. This review was conducted through a literature search in the ScienceDirect and PubMed databases. Inclusion criteria were full-text articles written in English and Portuguese that discussed the legal aspects, stress, management, challenges, and prospects of river transport of cattle in northern Brazil. As exclusion criteria, studies that did not align with the study’s objective were disregarded. The PRISMA checklist was used for conducting this review. The region relies on river transport due to road limitations, using rivers as the main logistical routes for moving products. The poor infrastructure of ports and vessels compromises animal welfare and highlights the need for investments to ensure efficiency and competitiveness in the production chain. River transport of cattle in western Para is an important component of Amazon livestock farming, being essential for product distribution and the integrity of the production chain. This activity involves challenges related to animal welfare, associated with adverse environmental factors, stocking density, long travel periods, and the constant movement of vessels. The implementation of good management practices, adequate infrastructure, and continuous monitoring strategies emerges as an effective way to reduce stress and prevent injuries during transport, along with technologies for monitoring and evaluating composite indices.

## Introduction

The river transport of cattle by means of rafts is widely used in the West of Pará, where the rivers function as the main access routes to production areas, slaughter centers and commercialization. This modality becomes indispensable due to the large territorial extension and the limitations of the road infrastructure (Bailone 2019; Gatti et al. 2021). However, prolonged displacement imposes important challenges to animal welfare, requiring careful evaluation to ensure the health of cattle and the quality of the final product (Silva et al. 2024a).

Long trips made by adapted boats or ferries can expose animals to heat stress, especially due to the high relative humidity of the region, temperature variations and often inadequate ventilation (Dunston-Clarke et al. 2020; Grandin 2019a; Grandin 2019b; Souza et al. 2026). These factors, associated with the high stocking density and prolonged confinement time, compromise thermal comfort and can trigger dehydration, weight loss, and stress-related physiological and behavioral changes (Hing et al. 2021; Silva et al. 2022a, 2024b).

Management during embarkation and disembarkation operations is another critical point, as the lack of appropriate infrastructure, the inadequate use of equipment, and the absence of low-stress management protocols favor the occurrence of injuries, abrasions, and fractures (Neto 2021). These damages harm welfare, reduce carcass quality, and generate economic losses for producers and slaughterhouses (Sousa et al. 2025).

Another relevant aspect is access to water and food during the trip. In long-term transports, the lack of adequate supply intensifies stress, compromises metabolism and increases the susceptibility of cattle to diseases. Similarly, the absence of monitoring and maintenance of the internal conditions of vessels such as ventilation, temperature, and humidity aggravates the effects of confinement and reduces productive performance (Buckham-Sporer et al. 2023; Schuetze et al. 2017).

In view of this scenario, the adoption of specific standards and good practices in river transport is essential to preserve the physical and physiological integrity of animals. Measures such as the use of appropriate vessels, correct definition of stocking density, continuous supply of drinking water, training of workers and monitoring of welfare indicators contribute to reducing stress and promoting the sustainability of livestock (Phillips 2008; Andrade et al. 2008; Masunga et al. 2025) in the region.

The transport of cattle by river in northern Brazil is an activity carried out throughout the year, especially during the rainy season, when roads become a challenge, as they become impassable due to heavy mud accumulation, which hinders the efficient transport of these animals. In this sense, river transport ends up being used as a fallback option, a strategy aimed at reducing potential damage or even waiting times for animals on roads due to broken-down trucks. However, there are few studies in this region on the effects of river transport on cattle welfare. Therefore, the objective was to conduct a systematic review of the legal aspects, stress, management, challenges, and perspectives of river transport of cattle in northern Brazil.

## Materials and methods

This systematic review was conducted following the PRISMA guidelines. A comprehensive literature search was performed in the ScienceDirect and PubMed databases, selected for their extensive coverage of peer-reviewed literature in animal science, veterinary medicine, and agricultural systems relevant to livestock transport and animal welfare.

The search strategy was developed from a combination of controlled keywords and free terms, using Boolean operators. The following search sequences were applied: (“river transport” and cattle), (“livestock transport” and rivers), (“cattle handling” and transport), (“animal welfare” and cattle transport), (“heat stress” and livestock transport), (“Amazon” and livestock transport), and (“Amazon region” and cattle transport). The searches were performed in the title, abstract, and keyword fields, including studies published up to October 2025, with no restriction on the year of publication.

The inclusion criteria comprised full articles written in English or Portuguese that addressed cattle transport in riverine or Amazon conditions, with direct or indirect assessment of animal welfare, stress factors, management practices, or environmental challenges associated with transport. Indirect or comparative studies (e.g., comparisons between transport modes or environmental conditions) were included when they provided relevant insights applicable to river transport in the Amazonian context. Studies were excluded if they did not align with the objective of assessing welfare-related outcomes in cattle transport or if they lacked sufficient methodological information.

The selection of studies was carried out in two stages. Initially, titles and abstracts were analyzed. Subsequently, the full articles were evaluated for eligibility. All screening steps were conducted independently by the authors, and disagreements were resolved by consensus to minimize selection bias.

The data extracted from the selected studies included the year of publication, the location of the study, the species and category of livestock (age, sex, or stage of production), transport characteristics, environmental conditions, experimental design, welfare indicators, and results related to stress and thermal comfort. In addition, the methodological quality of the included studies was assessed qualitatively based on the clarity of objectives, the adequacy of the study design, and the relevance of welfare indicators, allowing for a critical synthesis of the evidence rather than a purely descriptive summary.

**Fig. 1:**
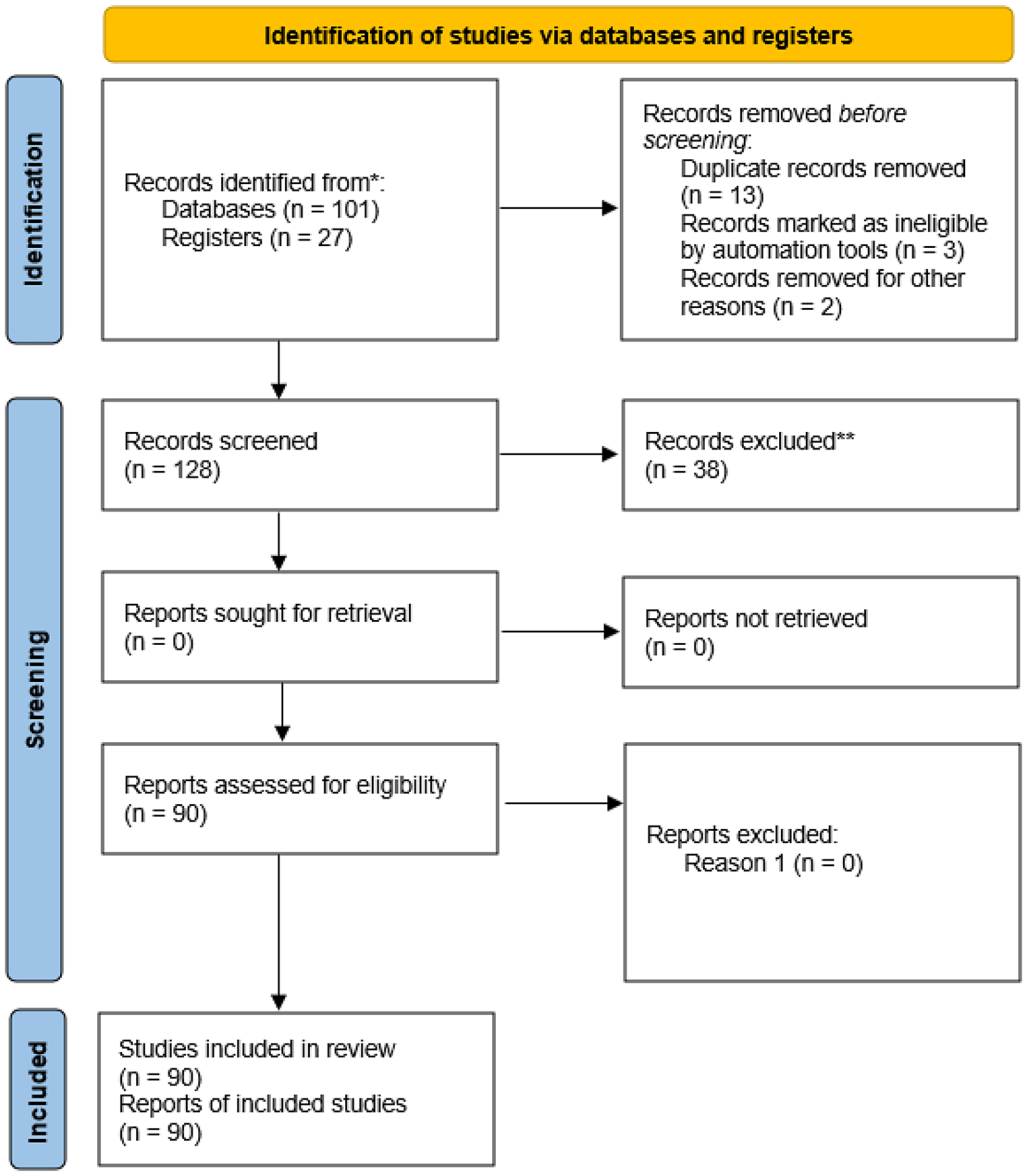
Flowchart illustrating the selection process of articles included in the systematic review, developed in Microsoft Word according to the PRISMA guidelines (2020). *Consider, if feasible to do so, reporting the number of records identified from each database or register searched (rather than the total number across all databases/registers). **if automation tools were used, indicate how many records were excluded by a human and how many were excluded by automation tools

All studies retrieved up to the end of the database searches were considered in the initial screening. A total of 128 records were identified, from which duplicate entries were removed and the remaining articles were screened based on titles and abstracts. After applying the inclusion and exclusion criteria and evaluating the full texts, 90 studies were selected and included in this systematic review, as detailed in the PRISMA flowchart (Fig. 1). The extracted data were organized in spreadsheets and subsequently analyzed, with the results presented in graphs and tables to facilitate data interpretation. The PRISMA checklist was used (Page et al. 2021).

Table 1 summarizes the experimental studies included in the review, presenting information on the authors, year of publication, study objectives, methods, and main results.

**Table 1:** Overview of experimental articles with empirical data considered in this review

Author	Year	Objective	Methods	Results
Acipar et al.	(2025)	Analyze territorial vulnerability and impacts of severe droughts in Tefé (AM)	Case study; analysis of environmental and socioeconomic data and interviews	Intense droughts increase social vulnerability, hinder regional circulation and directly affect local economic activities
Andrade et al.	(2008)	To evaluate the prevalence of injuries in cattle carcasses transported by waterways in the Pantanal	Post-mortem evaluation in a slaughterhouse; 789 carcasses inspection	High prevalence of injuries related to river transport; predominant bruises, indicating negative impact on welfare and meat quality
Bicalho et al.	(2015)	Analyze conflicts between rural communities and conservation units in the Central Amazon	Qualitative research with interviews, field observation and document analysis	The overlap between conservation areas and peasant territories generates management conflicts and limitations in the traditional use of natural resources
Caitano et al.	(2023)	Describe the technological profile of cattle ranching in Pará and discuss challenges of pasture sustainability	Quantitative research with secondary data analysis and interviews	Low technological adoption; degraded pastures are frequent; Sustainability depends on investments in management and training
Cortner et al.	(2019)	Evaluate perceptions about integrated crop-livestock systems in the Amazon for sustainable intensification	Semi-structured interviews with producers and rural agents	Producers recognize productive benefits, but cite barriers such as initial costs and lack of technical assistance
Dourado et al.	(2024)	To evaluate changes in land use and land cover associated with port dynamics in Santarém (PA)	Geospatial analysis, satellite imagery, and port expansion data	Expansion of the terminal resulted in an increase in urbanized areas and a reduction in vegetation in the region studied
Dunston et al.	(2020)	Establish a welfare assessment protocol for animals transported by sea	Methodological development; interviews with experts; Analysis of physiological and behavioral parameters	Structured protocol including environmental, physiological, behavioural and physical indicators, applicable to maritime transport
Džermeikaitė et al.	(2023)	Investigate innovative technologies and sensors applied to the diagnosis of diseases in cattle	Experimental study and application of sensors for physiological monitoring	Sensors provided early diagnosis of metabolic and behavioral disorders, improving accuracy and speed in management
Espinoza et al.	(2024)	Analyze the drought and heat records in the Amazon in 2023 and their regional and global climate causes	Climate modeling; analysis of historical series; Hydroclimatic data	2023 had the most severe drought on record; combination of strong El Niño and anomalous North Atlantic warming
Fernandes et al.	(2021)	Evaluate costs and benefits of on-farm animal welfare improvements	Economic analysis associated with management studies and productivity indicators	Better welfare reduces economic losses, increases productivity and improves animal health, compensating investments
Garcia et al.	(2017)	Assess the costs, benefits and challenges of sustainable cattle intensification in the Amazon	Economic analysis, interviews, and scenario modeling	Sustainable intensification reduces pressure for deforestation, but requires high investment and technical training
Gatti et al.	(2021)	Analyze the expansion of export cattle ranching in Pará focusing on logistical aspects	Documentary study, geographic data, interviews and historical analysis	Strong expansion linked to logistical improvements and increased port infrastructure; Significant territorial impacts
Grandin	(2021)	To evaluate how ease of management reduces pre-slaughter stress in cattle	Behavior observations in management systems and comparative analyses	Docile animals and suitable environments reduce falls, vocalizations and reactivity, reducing stress and economic losses
Hansen et al.	(2023)	Evaluate producers’ attitudes and barriers to the adoption of cow-calf contact systems	Semi-structured interviews with European producers	Recognized benefits, but there are barriers such as increased labor, logistics, and productivity concerns
Heiderscheit et al.	(2022)	To evaluate how long transport durations and water/feed deprivation affect the behavior of feedlot cattle	Behavioral observation of steers after prolonged transport, with recording of aggressive activities and interactions	Deprivation and prolonged transport increased abnormal behaviors and aggressive interactions in the trough
Ljungberg et al.	(2007)	Analyze the logistics of animal transportation and slaughterhouse operations	Evaluation of stages of the logistics chain using operational measurements, times and transport conditions	Logistical failures have been found to increase stress and the risk of injury during transport
Melasari et al.	(2025)	Evaluate the implementation of welfare standards on ships transporting live animals	Direct audits and assessments of transport conditions on livestock vessels	They found conformities and non-conformities in welfare standards, highlighting critical points in the structure of the ships
Grandson	(2021)	To evaluate the occurrence of bruises in bovine carcasses in Pará due to transport	Analysis of carcasses in slaughterhouses, quantifying types and frequency of bruises	He found a high occurrence of bruises, associated with inadequate handling and poor transport
North et al.	(2023)	Estimate the global risk of heat stress for cattle due to climate change	Climate modeling and environmental projections applied to cattle production	Forecasts show substantial increase in the risk of heat stress in several producing regions
Ramos et al.	(2021)	To investigate the relationship between occupational safety, occupational stress and adaptation of immigrant workers in cattle feedlots	Quantitative study with questionnaires applied to feedlot workers	High occupational stress was related to lower adaptation and perception of safety at work
Rodrigues-Junior et al.	(2021)	Assessing the water footprint of beef production in the Amazon biome	Survey of water consumption data throughout the production chain; calculation of the “water footprint” (liters of water per kg of meat produced) for beef cattle in the Amazon	The water footprint found was 13,074 L·kg^−^^1^ of meat—lower than the reported world average (~21,829 L·kg^−^^1^), indicating that cattle ranching in this Amazonian context can be relatively efficient in terms of water use
Rohleder et al.	(2022)	To evaluate environmental parameters (temperature, humidity) and their relationship with heat stress in dairy cattle in southern Amazonas (Humaitá-AM microregion)	Collection of environmental data: air temperature (TA) and relative humidity (RH) during dry and rainy seasons; calculation of the temperature-humidity index (THI); Comparative analysis between periods	THI values ranged from 56.25 (comfort) to 84.68 (emergence), demonstrating that even in times considered “milder” the combination of heat and humidity can lead cattle to levels of heat stress. Suggest shade use and management strategies to mitigate the impact on comfort and productivity
Sarubbi et al.	(2024)	To study hypothalamic neuromodulation and body surface temperature control in cattle during hyperthermia	Experiment evaluating physiological responses (surface temperature, neuromodulatory mechanisms) in cattle exposed to hyperthermia conditions; Measurement of thermal and regulation parameters	Results: evidence that hypothalamic neuromodulation acts to control skin temperature, which may influence management strategies to reduce heat stress in periods of intense heat
Silva et al.	(2024a)	To evaluate thermal comfort of Nellore cattle (Bos indicus) managed in silvopastoral and traditional systems in eastern Amazonia, associating with rumination	Field study with monitoring of thermal and behavioral parameters: collection of environmental data, observation of rumination, use of different breeding systems (silvopastoral vs traditional)	It demonstrated that silvopastoral systems offer better thermal comfort conditions, reducing heat stress and favoring natural behaviors such as rumination—pointing to the benefit of pastures with tree cover in tropical regions
Silva et al.	(2024b)	To evaluate the effect of transport distance and lairage on carcass behaviors and parameters in Zebu cattle	Experiment with focal sampling	It found that long transport and inadequate waiting time influenced stress behaviors and deterioration of carcass parameters—evidencing a negative impact on welfare and meat quality
Silva et al.	(2023)	Characterize thermal patterns using infrared thermography and thermolytic responses of cattle raised in three different systems during the transition period in eastern Amazonia	Use of infrared thermography to measure surface temperature; monitoring of thermolytic physiological responses; Comparison between rearing systems over the transition period	It identified significant differences in thermal patterns between rearing systems—systems with better management provided more effective thermoregulatory responses, suggesting that management and environment directly affect the thermal comfort of the animals
Sullivan et al.	(2024)	To evaluate pre-slaughter factors that influence mobility, blood parameters, bruising and muscle pH of finished cattle	Observational study on a farm/slaughterhouse in the USA; pre-slaughter management data collection, blood tests, bruise assessment, and post-slaughter pH measurement	It found that pre-slaughter practices strongly impact the mobility of animals, increase the incidence of bruises and alter muscle pH—resulting in damage to meat quality and animal welfare
Schuetze et al.	(2017)	Investigate the transport of commercially finished cattle and animal welfare considerations	Study with transport monitoring, well-being assessment with indicators of stress, mortality, post-transport injuries	It identified that commercial transport, especially on long journeys, can compromise well-being and increase the risk of injury and mortality, recommending more careful handling and transport practices
Shin et al.	(2022)	To evaluate the applicability of a demand-controlled ventilation system in cattle breeding facilities	Experiment in a confinement environment using a demand-controlled ventilation system; Measurement of environmental and animal welfare parameters	The system showed efficiency in improving thermal comfort and reducing heat stress, suggesting it as a tool for management in tropical or hot climates
Uchendu et al.	(2022)	To assess the impact of road transport on physiological biomarkers and oxidative stress – albeit in dogs (but as a comparative effect of transport)	Collection of pre- and post-transport physiological data and analysis of oxidative stress biomarkers and welfare parameters	Road transport caused significant changes in biomarkers, showing that transport stress affects domestic animals – a methodological reference that can be adapted for cattle
Valadez et al.	(2022)	To evaluate the consequences of long-distance transport on the behavior and health of young steers, aiming at their adaptation to feedlots	Field study with behavioral monitoring and health parameters before and after long transport; Feedlot Adaptability Assessment	Prolonged transport resulted in behavioral changes, decreased health and less adaptation to confinement, which can compromise performance and well-being
Válková et al.	(2021)	To assess the welfare of cattle, sheep, goats and pigs from traumatic injuries detected in postmortem slaughterhouses.	Collection of post-slaughter inspection data from carcasses; injury and contusion records; comparative analysis between species.	It demonstrated that a significant proportion of the animals had traumatic injuries, signaling failures in pre-slaughter management and transport, with serious implications for animal welfare
Vlaicu et al.	(2024)	Propose smart livestock systems to improve productivity, welfare and sustainability of animal production	Development and evaluation of integrated management technologies and systems with automation, environmental monitoring and welfare control	The proposed system pointed to increased production efficiency, stress reduction, and better welfare monitoring—showing the potential of technologies for sustainable management
Wilmann et al.	(2020)	To evaluate the effect of the duration of rest-stops during long-distance transport on cattle welfare indicators	Experiment with cattle submitted to long-term transport with different stoppage durations; Assessment of well-being via physiological and behavioral indicators	Longer rest-stops reduced stress markers and improved well-being—suggesting that strategic pauses in transport can mitigate negative impacts
Zanardi et al.	(2022)	To investigate the relationship between bruises in cattle carcasses and factors related to transport	Research with analysis of slaughtered carcasses: injury record, cross-referencing with transport data (distance, time, condition)	It found a significant association between transport and incidence of bruises on carcasses, reinforcing the negative impact of inadequate transport on meat welfare and quality

Note: THI = temperature-humidity index

## Literature review

### Contextualization of cattle ranching in western Pará and the importance of river transport

In Western Pará, cattle ranching represents one of the main economic activities, playing a strategic role in the supply of meat to national and regional markets, in addition to contributing to income generation (Caitano et al. 2023). In this context, the municipalities of Santarém, Óbidos, Alenquer and Monte Alegre, have beef cattle breeding mostly in an extensive system, standing out in the sector, which favors them by the availability of natural pasture areas and traditional livestock management (Silva et al. 2023, 2024b). This activity boosts the region’s economy, generates jobs directly and indirectly, and consolidates cattle ranching as an important pillar of agricultural production in the Amazon (Caitano et al. 2023).

The territorial characteristics of the region stand out for its vast floodplains and an extensive hydrographic network, composed of large rivers, such as the Amazon, and important tributaries, such as the Tapajós and the Trombetas. These waterways go beyond their natural function, assuming an essential role in the regional logistics infrastructure. During the flood season, rivers function as true highways, enabling the transport of large volumes of cargo, including animals, with greater energy efficiency and reduced costs compared to exclusively road transport (Leão et al. 2023).

Despite the presence of road transport, road infrastructure is limited, and many roads have maintenance problems, aggravated during the rainy season, when land access, in some cases, becomes unfeasible. In this context, river transport is a more efficient alternative for the flow of cattle production, ensuring the connection between rural properties and slaughter, processing, and marketing centers, located in urban areas or in other states (Neto 2021).

**Fig. 2:**
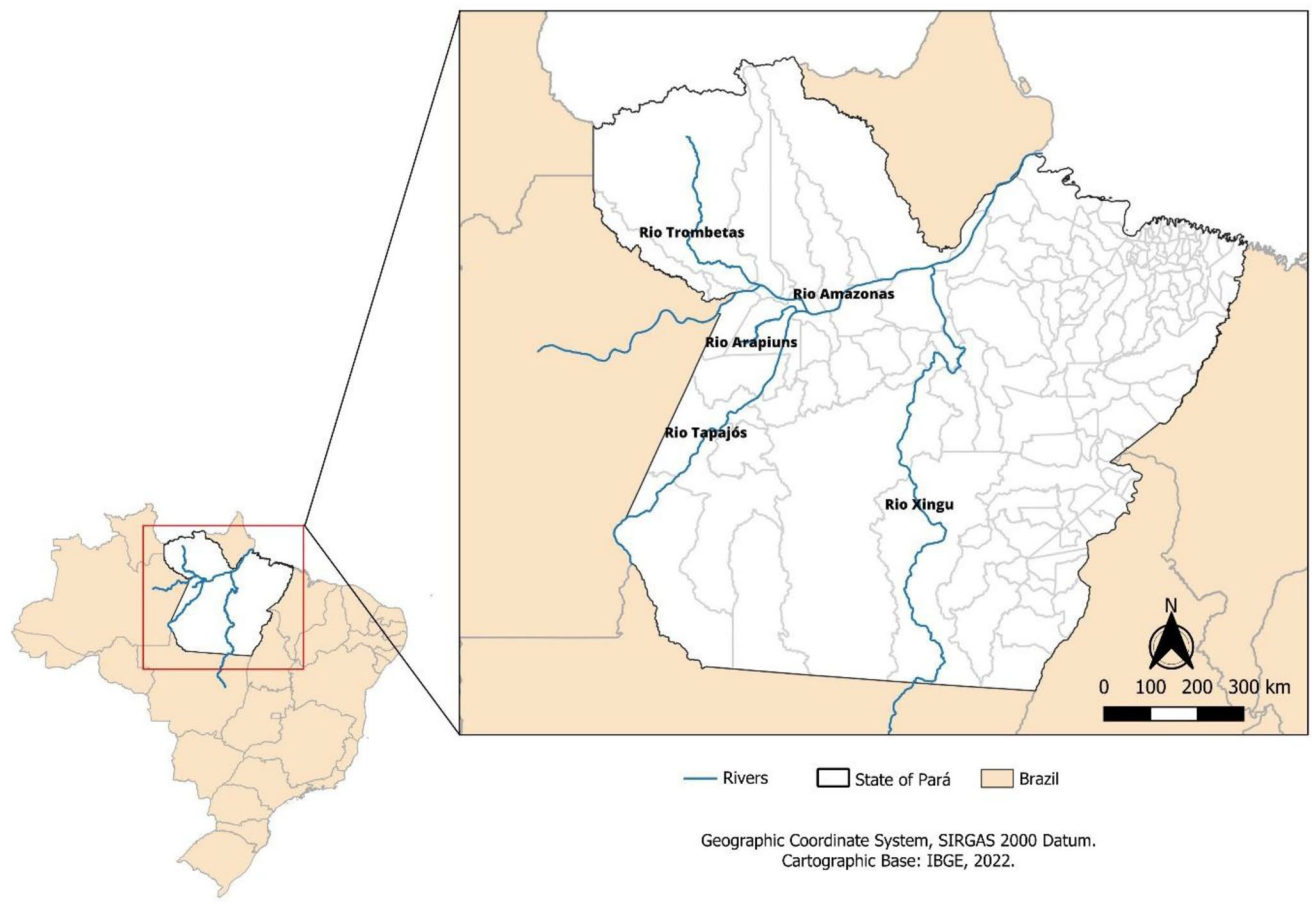
Main rivers used for cattle transport via inland waterways in western Pará. Figure developed in Canva. Source: personal archive

Rivers are consolidated as the main logistics routes (Fig. 2), not only because of the region’s territorial extension but also because of the cost-benefit of navigation. Transport by ferries and adapted vessels allows the movement of large batches of animals in a single trip, with lower fuel consumption compared to road transport. This efficient characteristic contributes to the competitiveness of the beef production chain, making river transport a central element of livestock in Western Pará (Fleming et al. 2020).

The dependence on river transport in the region shows important structural limitations. In several stretches of navigation, the absence of adequate ports compromises the safe movement of cattle, as well as appropriate loading and unloading structures that meet animal welfare requirements. This lack of adequate infrastructure increases the possibility of risk of accidents, can cause injuries to animals and hinder the adoption of rational management practices during transport (Júnior et al. 2011; Silva et al. 2022a, b).

Another critical aspect refers to the conditions of the vessels used in transport. In several cases, boats and ferries were not originally designed for the transport of animals, requiring improvised adaptations that compromise the well-being of cattle, especially with regard to comfort, ventilation, and safety (Bailone 2019) This inadequacy favors the occurrence of heat stress and increases the susceptibility of animals to injuries, directly impacting the quality of meat and the productivity of the livestock chain (Ludolf and Costa 2020a, b).

In addition, the seasonal hydrological regime of Amazon rivers exerts a direct influence on transport logistics. During the ebb season, the reduction in water levels makes it difficult to navigate certain stretches, requiring rigorous planning to avoid delays or interruptions in the movement of the herd. This natural dynamic imposes the need to adopt flexible flow strategies, capable of aligning the production calendar with navigability conditions (Leão et al. 2023).

The dependence on river transport is related to the socioeconomic conditions of local producers. Most rural properties are far from paved highways and do not have adequate infrastructure for land flow. In this context, for these producers, rivers assume a role that goes beyond a simple means of transport, functioning as an essential element of integration with consumer markets, ensuring the commercialization of the herd and the economic sustainability of families that have livestock as their main source of income (Neto 2021).

Therefore, understanding the panorama of cattle farming in Western Pará and the strategic role of rivers as the main logistical axes becomes fundamental for the planning of public policies and for directing investments in infrastructure (Bailone 2019). The search for improvements in ports, the adaptation of vessels, and the training of workers can not only increase the efficiency of transport, but also minimize the impacts on animal welfare, strengthening regional livestock and ensuring the competitiveness of Amazon meat in domestic and foreign markets (Lourenço et al. 2020).

### Legal and normative aspects of animal transport in Brazil

**Fig. 3:**
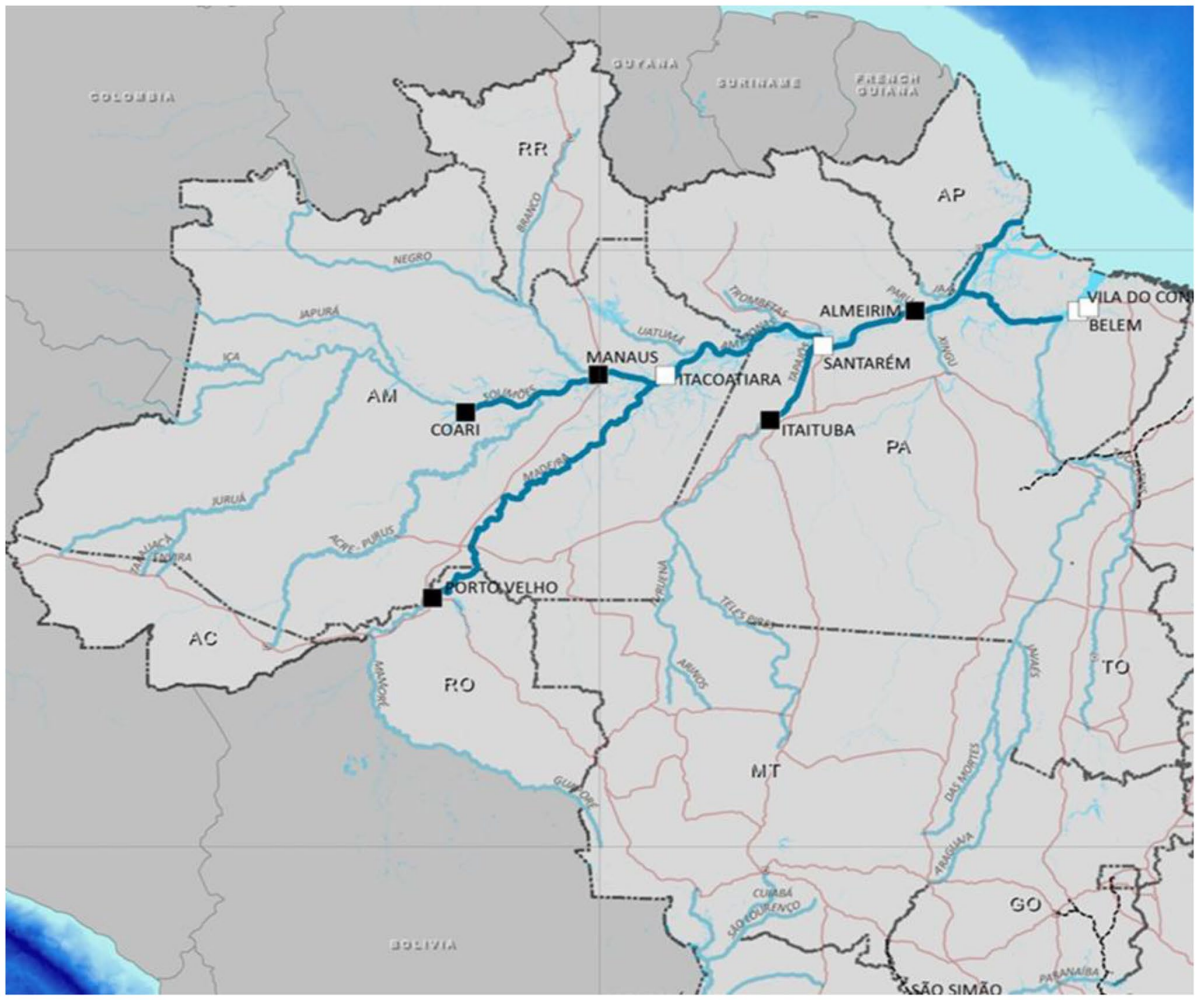
Main outbound transport routes in the western region of Pará. Figure developed in Canva. Source: adapted from Brazil (2013)

The transport of live animals in Brazil is regulated by laws, decrees, and normative instructions that aim to ensure both food safety and animal welfare (Tavares and Neto 2025). These standards are based on scientific and ethical principles, seeking to reduce stress and physical damage during commuting. In the case of river transport of cattle, this care becomes even more relevant in regions such as the West of Pará, where the river is the main route for the flow of livestock production. The Fig. 3 depicts the main transport routes (Dias-Filho and Lopes 2020).

Among the main legal frameworks is Law No. 11,794/2008, which establishes guidelines on the use of animals in teaching and research, defining general welfare principles that influence other standards. Applied to the livestock sector, MAPA’s Normative Instruction No. 56/2008 establishes parameters for land transport, such as cargo density, water supply and management in loading and unloading. Although focused on road transport, this regulation also serves as a reference for river transport, due to the absence of specific regulations for this type of operation in the Amazon (Silva et al., 2022a; Rohleder et al. 2022).

Another point of importance is Decree No. 9,013/2017, which regulates RISPOA, establishing responsibilities for the transporter and the producer on the conditions of animals destined for slaughter (Fernandes et al. 2021). Requiring that they arrive without injuries that compromise the quality of the meat. In view of this, even indirectly, RISPOA reinforces the need for good practices in river transport to maintain the standards required by the consumer market (Costa 2024).

National legislation is also influenced by international standards, such as those of the World Organization for Animal Health (WHOA), which establishes global welfare parameters during transport. These recommendations include the need to avoid heat stress, ensure sufficient space, water, and food, guidelines that apply to the Amazon reality (Nielsen et al. 2022). However, adapting these precepts to the daily life of river transport still represents a major challenge (Rohleder et al. 2022).

Despite the general rules, one of the main obstacles to the transport of cattle by rivers is the absence of specific regulations. The instructions currently in force were designed for road transport and do not consider the particularities of the waterway, such as variations in river levels, long distances, and the need for adapted vessels (Lima et al. 2024). These aspects require their own rules, which are not yet contemplated in laws, normative instructions or decrees (Acipar and Queiroz 2025).

The lack of detailed regulation generates legal uncertainty and limits inspection. Many vessels do not follow technical standards for accommodation, ventilation or water supply, which can increase the stress of the animals and cause economic losses. In addition, the absence of official criteria makes it difficult to standardize good practices and certify quality, harming the competitiveness of Amazon livestock in the face of a market that increasingly requires proof of animal welfare (Zanardi et al. 2022; Costa 2024).

The training of inspectors and transport agents is another critical point in this context. Inspection agencies often do not have sufficient human and structural resources to monitor these operations in ports and vessels. This reinforces the need for specific policies that consider the Amazon reality and offer better conditions for inspection and training (Dourado et al. 2024; Espinoza et al. 2024).

The creation of specific regulations for river transport should include both technical requirements for vessels and handling protocols during embarkation, travel, and disembarkation (Silva et al. 2022a). It is essential to define criteria for animal density, ventilation, maximum travel time, access to water and food, as well as welfare indicators to be monitored. Such measures would provide greater legal certainty and raise the quality of meat produced in the region (Nielsen et al. 2022).

Although Brazil has a set of rules aimed at animal welfare, there are still gaps in the regulation of river transport of cattle in the West of Pará. Specific standards, adapted to the reality of the region and in line with international recommendations, are essential to ensure the sustainability of livestock, the protection of animals during displacement, and strengthen the country’s presence in markets that value responsible and ethical practices (Fernandes et al. 2021; Costa 2024).

### Stress factors during river transport

The river transport of cattle in western Pará presents several challenges that can negatively affect the well-being of animals, especially on long trips and with inadequate handling. Among the main stress factors are the typical environmental conditions of the Amazon region, overcrowding on vessels, the duration of the journey, and the constant movement of boats during navigation (Silva et al. 2022b). These factors interact, increasing the risk of discomfort, injuries, and physiological imbalance (Silva et al. 2022b).

Environmental conditions represent one of the greatest challenges for the preservation of animal welfare. The Amazon region is characterized by high relative humidity, often above 80%, and average temperatures ranging between 25 and 32 °C (Silva et al. 2024c; Rohleder et al. 2022). In vessels with poor ventilation, these factors favor heat stress, making it difficult for cattle to dissipate body heat. The combination of insufficient ventilation and inadequate spaces intensifies thermal discomfort, which can cause hyperthermia and dehydration (Silva et al. 2024a).

Inadequate ventilation is a critical factor. Ferries and boats adapted for cattle transport do not offer sufficient openings to ensure effective air circulation (Nielsen et al. 2022). This problem is aggravated in situations of high density of animals, since the metabolic heat generated contributes to raising the internal temperature, creating an adverse microclimate. Excess heat and humidity make it difficult for cattle to thermoregulate, increasing respiratory rate and heart rate (Silva et al. 2023).

Stocking density is another relevant factor for heat stress. When the number of animals transported exceeds the ideal capacity of the vessel, the risks of trampling, falls, and injuries increase (Rebouças et al. 2021; Silva et al., 2022a). The reduced space compromises the mobility of the cattle, preventing them from adjusting to maintain balance during navigation. This confinement, combined with constant physical contact, generates agitation and social conflicts, raising cortisol levels and other physiological markers of stress (Abubakar et al. 2021).

**Fig. 4:**
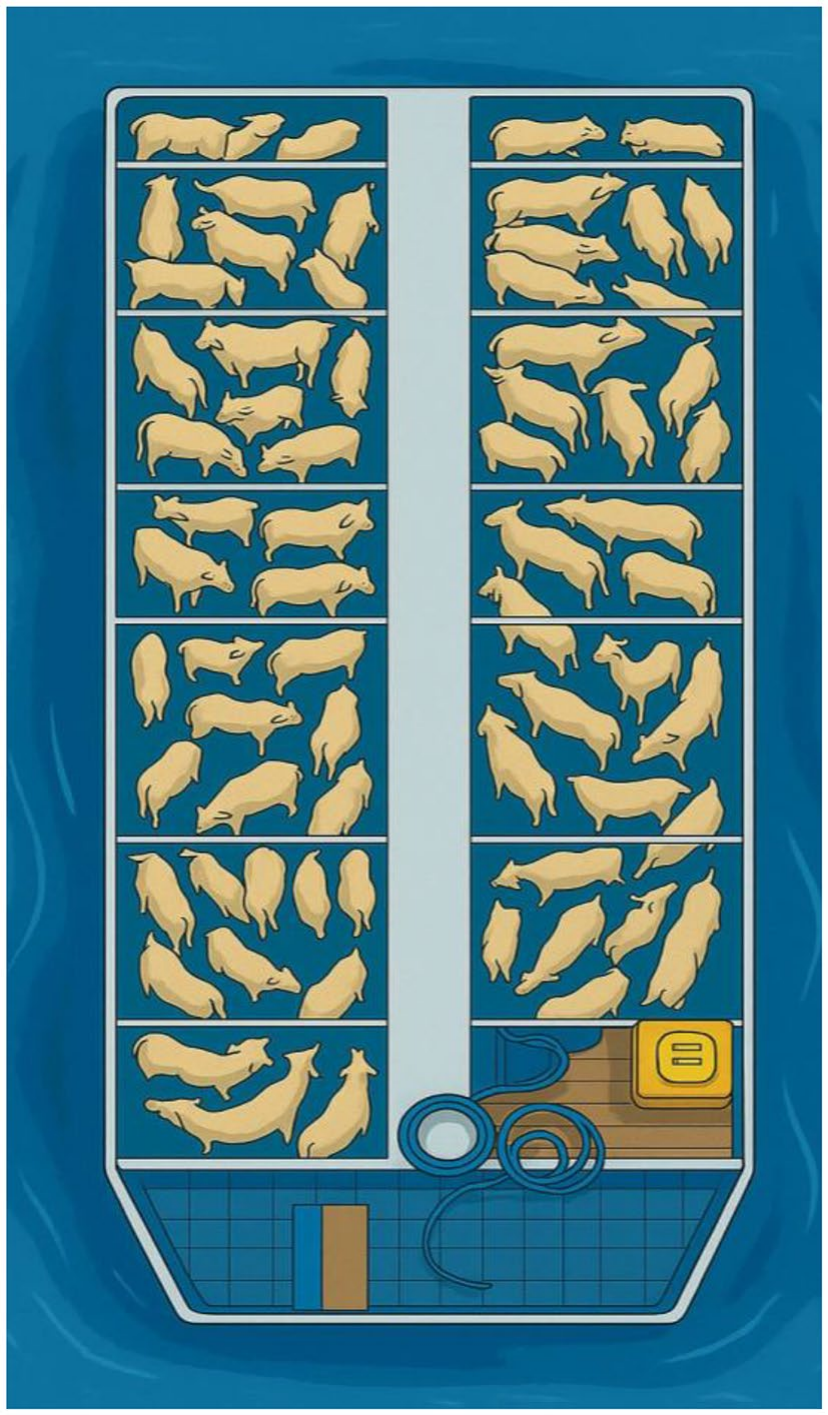
High stocking density in river vessels used for cattle transport in the Amazon, where animal crowding in confined spaces compromises welfare and increases the risk of falls, injuries, and physiological stress. Figure developed in Canva. Source: personal archive

Figure 4 illustrates the high stocking density of a river vessel used to transport cattle in the Amazon. The agglomeration of animals in a reduced space is observed, which limits freedom of movement, increases the risk of falls and favors situations of physiological stress. This scenario reinforces the importance of cargo planning and the adoption of animal welfare criteria during waterway transport.

The duration of the journey has a great influence on the welfare of the animals. On journeys that can last several hours or even days, the absence of rest breaks and the insufficient supply of water and food intensify dehydration (Heiderscheit et al. 2022). In addition, the continuous noise of the engines and the vibrations of the vessel’s hull contribute to the physical and emotional wear and tear of cattle, affecting both their behavior and physiological functioning (Brasil 2021).

The very movement of the vessel imposes additional challenges on the animals. Oscillations caused by waves, variations in current, and sudden maneuvers require cattle to constantly adjust to maintain balance (Ludolf et al. 2022). These continuous efforts, added to the risk of slipping on unsuitable floors, can cause muscle strain, bruises and even fractures (Phillips et al. 2013; Lilienthal et al. 2019). On night trips, low visibility aggravates these risks, requiring greater care in the handling and structure of the floor.

Physiological effects in these conditions include changes in animal parameters, such as increased respiratory and heart rate, increased body temperature, and reduced water and food consumption (Uchendu et al. 2022). These factors affect energy metabolism and immune response, increasing susceptibility to diseases and compromising the productive performance of cattle (Valadez et al. 2022).

Cattle under stress show changes in behavior, signs such as restlessness, excessive vocalizations, aggressiveness, and an attempt to escape (Ludolf and Costa 2020a). These behaviors indicate suffering and make safe management difficult, increasing the risk of accidents for both animals and workers (Buckham-Sporer et al. 2023). Close observation of these signs is essential for quick and effective interventions during the trip.

The combination of factors such as a hostile environment, overcrowding, prolonged travel time, and constant vessel movement makes river cattle transport a significant challenge for welfare (Nielsen et al. 2022). Recognizing these stressors is critical to implementing mitigation strategies, such as improvements in ventilation, adjustment in load, adequate water and food supply, continuous monitoring of thermal comfort and health indicators (Buckham-Sporer et al. 2023).

### Pre-shipment, embarkation and disembarkation handling

Proper management of cattle before, during, and after river transport is essential to ensure animal welfare and minimize economic losses (Grandin, 2019a; Nielsen et al. 2022). The process leading up to boarding, including the preparation of animals and facilities, influences stress levels and the incidence of injuries during the journey (Zanardi et al. 2022). In the West of Pará, where waterway transport is the main means of transporting production, the adoption of good management practices at all stages is essential for the sustainability of regional livestock.

Pre-boarding requires special attention. Animals should be kept in suitable waiting pens, with continuous access to clean water and light feeding (Sullivan et al. 2024). This rest period contributes to physical recovery, especially after land travel, and helps stabilize physiological parameters, reducing stress levels before the trip (Heiderscheit et al. 2022). At this stage, sick, injured, or debilitated cattle should be identified and separated for veterinary evaluation, avoiding their transport in conditions that could further compromise their health or cause suffering (WOAH 2021).

Boarding represents one of the most sensitive stages of the process, as it involves intense movement and a higher risk of injury (Nielsen et al. 2022). Cattle should be driven calmly, avoiding screaming, aggression or the use of objects that cause pain, such as electric batons. Low-stress methods such as the use of flags, rattles, and proper body language are highly recommended (Garcia et al. 2017).

The lighting of the boarding area must be sufficient to ensure that the animals can clearly see the route, minimizing stops and crowds (Grandin 2021). In addition, the access structure to the vessel must value safety, with ramps with an inclination of less than 20°, a width compatible with the size of the animals and lateral handrails to assist in driving (Brasil 2019). The floor should be non-slip, using materials such as ripped wood or rubber, to prevent slips, falls, and fractures (Grandin, 2019b).

During the journey, continuous monitoring of the vessel’s internal conditions is essential. Temperature, ventilation, and humidity must be controlled to avoid heat stress and the accumulation of gases (Melasari et al. 2025). Whenever possible, it is necessary to offer drinking water to the animals and, on long trips, to offer adequate fibrous food. Such care helps prevent dehydration, weight loss, and a drop in productive performance (Wilmann et al. 2020).

**Fig. 5:**
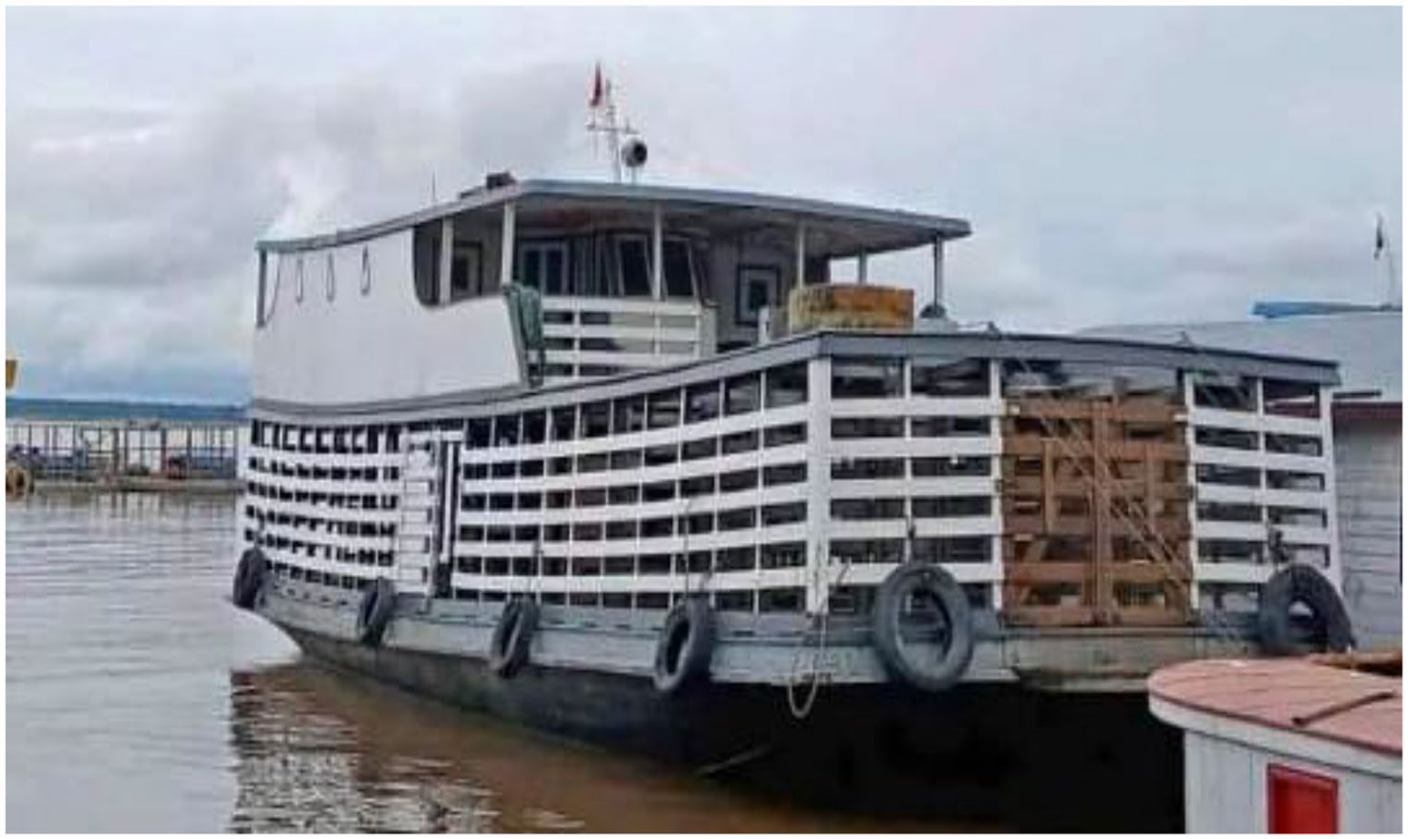
Vessels used for the inland waterway transport of cattle. Source: personal archive

As illustrated in Fig. 5, the vessels used in the river transport of cattle in the Amazon often operate in open environments, with direct exposure to climatic variations. This configuration, typical of regional navigation, imposes additional challenges to the control of the internal microclimate, especially on days of intense heat or high humidity. The absence of shade and limited natural ventilation can aggravate heat stress, reinforcing the need for preventive measures and continuous monitoring (Melasari et al. 2025).

The disembarkation stage must be carried out with the same care as boarding, in a calm and orderly manner. Gently sloping exit ramps, non-slip flooring, and side barriers are key to preventing accidents and facilitating the flow of animals (Grandin, 2022). It is important to avoid rushing or coercive methods, which increase the risk of injury and cause additional stress (Nielsen et al. 2022).

Soon after landing, it is recommended to inspect the cattle, with the aim of identifying possible injuries, signs of exhaustion, or behavioral changes (Valkova et al. 2021). Early detection of these problems allows for the adoption of immediate corrective actions, avoiding future losses (Buckham-Sporer et al. 2023).

One of the pillars for the success of management is the training of the workers involved. Everyone should receive training in animal welfare, focusing on low-stress driving techniques, recognition of signs of discomfort, and emergency responses (Brazil 2021). Valuing animal welfare as an ethical and economic aspect strengthens the responsibility of workers and improves the image of the sector.

Additionally, it is essential to establish standardized operating protocols. They must cover all phases of transport, from planning the capacity per trip to cleaning and disinfecting the management areas. The standardization of practices facilitates inspection, promotes safety, and ensures the adoption of procedures based on scientific evidence (Ludolf et al. 2022).

**Fig. 6:**
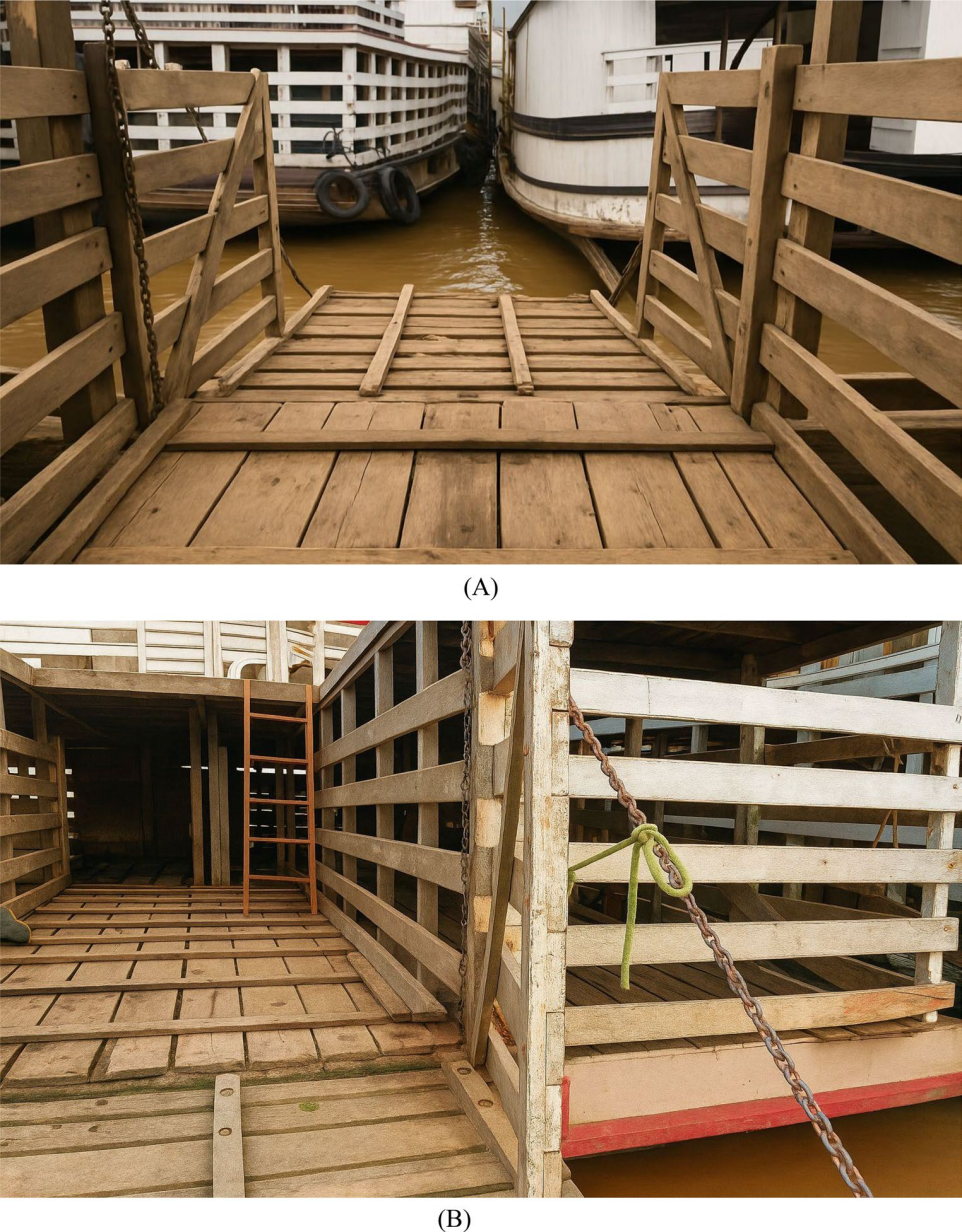
Image of the entrance ramp (**A**) and descent of the animals (**B**). Source: personal archive

Figure 6 (A and B) illustrates the route used by cattle during boarding and disembarkation on the ferry. River transport in the Amazon requires adequate infrastructure, appropriate and humane handling practices, and a trained workforce. The adoption of ramps with a safe slope, non-slip floors, control of environmental conditions, and continuous training of the team are indispensable strategies to prevent and minimize physiological stress, and ensure the well-being of the animals throughout the process (Hansen et al. 2023).

More than meeting legal requirements, these measures result in concrete benefits for the production chain, as they reduce economic losses, improve meat quality and increase operational efficiency.

### Strategies to ensure well-being during river transport

The conditions of cattle transport in Western Pará require specific strategies that consider both the environmental characteristics of the Amazon and the logistical challenges of navigation (Martorano et al. 2021). The long duration of the trips, added to the variations in temperature and humidity, reinforces the need for detailed planning and constant monitoring, in order to preserve the health and comfort of the animals (Papakonstantinou et al. 2024). The integration between good management practices and the monitoring of environmental and physiological parameters is essential to reduce stress, avoid production losses, and strengthen the sustainability of the meat chain (Rebezov et al. 2024).

Adequate water and feed supply forms the basis of animal welfare during transport (Nielsen et al. 2022). Continuous hydration is essential to prevent dehydration, especially in a hot and humid climate like the Amazon (Santos et al. 2025). To this end, it is recommended to install drinking fountains or drinking water supply systems at strategic points on the vessels, ensuring easy access (Rodrigues-Junior and Dziedzic 2021).

On trips longer than 12 hours, the inclusion of fibrous foods – such as good quality dry forage – helps maintain rumen function, reduces anxiety caused by food deprivation and contributes to physiological stability, in addition to minimizing weight loss at the end of the journey (Hogan et al. 2007).

Environmental control is also decisive in minimizing heat stress (Shephard and Maloney 2023). Regular measurement of temperature and relative humidity, using thermometers and hygrometers, enables rapid interventions whenever critical values are reached (Islam et al. 2021). Adjustments in ventilation and stocking density are applicable measures to avoid hyperthermia and ensure thermal comfort (Toledo et al. 2022). This preventive monitoring allows for real-time corrective actions, reducing risks to the health of animals (Džermeikaitė et al. 2023).

Adequate ventilation is another indispensable requirement (Shin et al. 2022). Vessels must have wide side openings or mechanical systems that favor air circulation, avoiding the formation of hot microclimates and the accumulation of gases (Schuetze et al. 2017).

During periods of higher humidity or during night trips, it is essential to maintain efficient air renewal, as high humidity compromises evaporative heat dissipation, increasing susceptibility to hyperthermia (Sarubbi et al. 2024). A well-planned ventilation system improves thermal balance and reduces respiratory stress (Emmanuel et al. 2025).

Continuous observation of animals through welfare indicators is equally essential (Keeling et al. 2021). Evaluating physiological parameters, such as respiratory rate and body temperature, in addition to monitoring water consumption, allows early identification of signs of discomfort (Idris et al. 2021). Behaviors such as intense vocalization, excessive salivation, tremors, or prostration indicate the need for immediate intervention (North et al. 2023). Checking for injuries, bruises, or falls also helps to detect handling failures or structural problems on the vessel (Zanardi et al. 2022).

The use of remote monitoring technologies – such as temperature and humidity sensors connected to alarm systems – represents a breakthrough for transport management (Mishra and Sharma 2023). These features reduce the exclusive reliance on manual inspection and enable faster and more accurate decision-making in the face of critical environmental variations (Montalván et al. 2024). Although still little explored in the Amazon, such innovations can become strategic differentials for the sector (Akhigbe et al. 2021).

Another decisive factor is the training of the crew and management team (Phillips 2024). Workers trained in observation techniques, identifying signs of stress, and responding quickly to emergencies make the process safer and more efficient (Ramos et al. 2021). Continuous training contributes to consolidating a culture of responsibility and respect for animal welfare, an aspect increasingly valued by consumers and international markets (Silva et al. 2024c).

It is important to highlight that the adoption of these practices positively impacts not only the well-being of cattle, but also the quality of the final product (Hansen et al. 2023). Animals transported in better conditions arrive at their destination with a lower level of stress, with better carcass yield, lower incidence of bruises, and adequate meat pH – characteristics that add commercial value and increase the competitiveness of regional livestock (Singh et al. 2024).

The promotion of animal welfare during river transport depends on the integration of different measures: continuous supply of water and food, environmental and physiological monitoring, efficient ventilation, use of remote monitoring technologies, and staff training (Dunston-Clarke et al. 2020). The coordinated application of these strategies ensures more ethical, safe, and economically sustainable transport, meeting current requirements for quality, social responsibility, and environmental conservation (Cislaghi et al. 2023).

### Advantages and disadvantages of river cattle transport in western Pará

The river transport of cattle in the Amazon plays a crucial role in the logistics of regional cattle ranching, especially in areas of difficult access, such as the West of Pará. With a vast network of navigable rivers, the region depends on this modality for the flow of large volumes of animals destined for slaughter (Tavares and Neto 2025; Dias-Filho and Lopes 2020). However, this means of transport has both advantages and disadvantages, directly impacting animal welfare and the efficiency of the logistics process (Silva et al. 2022b; Nielsen et al. 2022).

One of the main advantages is the reduction of operating costs. Studies indicate that river transport can be up to four times cheaper than road transport, due to the high load capacity of vessels and lower fuel consumption (Dourado et al. 2024; Tavares and Neto 2025). In addition, the lack of costs with the construction of roads, since rivers are natural routes, also contributes to the economy. This reduction in operating costs is crucial for cattle ranchers in the region, where logistics expenses can represent a significant portion of total expenses (Dias-Filho and Lopes 2020).

In addition, river transport has a lower environmental impact compared to road transport. Larger vessels can transport more tons of products with proportionally lower fuel consumption, reducing the emission of polluting gases (Lima et al. 2024). This aspect makes this modality a more sustainable alternative for the transport of production in the Amazon. However, it is important to emphasize that the lack of specific regulation for transport in the region can compromise these environmental benefits if inadequate practices are adopted (Acipar and Queiroz 2025; Espinoza et al. 2024).

However, the river transport of cattle faces significant challenges. The absence of specific regulation for this modality in the Amazon region is one of the main obstacles. The existing standards were developed for road transport and do not consider the particularities of river transport, such as the variation in the level of rivers and the need for adapted vessels (Acipar and Queiroz 2025; Tavares and Neto 2025). This regulatory gap makes it difficult to implement good practices and adequate inspection, compromising animal welfare and the quality of the meat produced (Silva et al. 2022a).

In addition, environmental factors, such as drought, can negatively affect river transport. The lack of rainfall can reduce the level of rivers, making navigation difficult and increasing the risk of vessel strandings (Lima et al. 2024). This phenomenon can compromise the safety of animals and the efficiency of transport. Climate variability requires flexible and adaptable logistics planning, as well as investments in infrastructure that allow continuous operation during different seasons of the year (Espinoza et al. 2024).

The lack of adequate infrastructure is also a significant limitation, although rivers are widely used, port infrastructure and specialized vessels are insufficient to ensure animal welfare during transport. The absence of adapted vessels and adequate port terminals can significantly increase the stress of animals and compromise of products from these animals (Rohleder et al. 2022, Silva et al. 2022a; Dourado et al. 2024).

In Table 2 it is possible to verify the advantages, disadvantages and limitations of the vessels.

**Table 2:** Advantages, disadvantages and limitations of river transport in the West of Pará

Information	Advantages	Disadvantages	Limitations
Logistics	Reduces transportation costs over long distances compared to road transport.	It depends on weather conditions and river levels, which can vary seasonally.	Insufficient port infrastructure in many locations.
Animal stocking	It allows the transport of large batches of animals in a single trip.	Poorly adapted vessels can increase the risk of injury.	Need for specialized and high-investment vessels.
Operational cost	Fuel and maintenance generally cheaper on extensive river routes.	Additional costs with pre-shipment management and welfare monitoring.	Dependence on skilled labor and constant training.
Disposal of animals	It enables the transport of production in regions that are difficult to access by land.	Interruption in periods of drought or extreme flooding can delay delivery.	Little integration with other logistics modes in some areas.
Animal welfare	Possibility of adapted boats with natural ventilation and adequate space.	Unadapted vessels increase the risk of hyperthermia, dehydration and bruises.	Continuous monitoring of temperature, humidity and physiological indicators is still not widespread.

### Challenges and perspectives for the sustainability of Amazonian livestock

Cattle ranching developed in the Amazon, especially in the West of Pará, faces relevant obstacles in terms of sustainability, involving environmental, economic, and social dimensions (Garcia et al. 2017). The river transport of cattle, a central element of this production chain, represents both a logistical solution and a point of vulnerability, especially when animal welfare does not receive due attention (Fike and Spire 2006). The stress generated by long trips and in adverse conditions compromises productivity, meat quality and the image of the sector, requiring integrated strategies that reconcile efficiency, ethics and environmental conservation (Damtew et al. 2018).

The economic impacts of animal stress are broad (Fernandes et al. 2021). Animals subjected to external displacements suffer weight loss, reduced carcass yield, and a greater occurrence of injuries and bruises – factors that affect the competitiveness of the product (Zanardi et al. 2022). In addition, changes in the pH of meat can lead to defects such as PSE (pale, soft, and exudative) or DFD (dark, firm, and dry) meat, reducing its market value and acceptance by consumers (Sristi et al. 2025). Thus, failures in management during transport result in direct and indirect losses for both cattle ranchers and slaughterhouses (Costa 2024).

To overcome these challenges, the adoption of technologies and innovations is essential (Abdul-Majid et al. 2024). The use of temperature, humidity, and air quality sensors, automated food and water supply systems, and remote monitoring tools allow for rapid interventions that minimize stress and mortality risks (Džermeikaitė et al. 2023). The structural adaptation of vessels with non-slip floors, efficient ventilation and adjustable compartments also contributes to reducing the negative impacts of river transport (Reyes 2024).

Another crucial point is the articulation between good management practices, public policies and certifications (Sims et al. 2005). The creation of specific standards for the transport of animals in the Amazon, in line with international welfare guidelines, can ensure greater standardization and legal support (Reis and Molento 2020). At the same time, certifications that value properties and carriers committed to sustainable and low-stress practices add value to the product and expand access to markets that are more demanding in socio-environmental terms (Bicalho and Hoefle 2015).

The continuous training of producers and workers represents another pillar of this transformation (Phillips 2024). Training on low-stress management, identification of signs of discomfort, and proper animal management are essential to reduce failures and enhance the use of new technologies (Ramos et al. 2021). In addition, investment in supporting infrastructure, such as safe ports and boarding ramps, strengthens operational efficiency and decreases the risk of injury to animals (Reyes 2024).

The sustainability of Amazon livestock depends on an integrated approach, capable of aligning profitability, respect for animals, and environmental conservation (Cortner et al. 2019). The river transport of cattle, when carried out in a planned and responsible manner, can cease to be a bottleneck and become a competitive differential (Ljungberg et al. 2007). The combination of technological innovations, specific regulation, certifications, and human training points to a promising path, in which regional livestock is consolidated as ethical, productive, and sustainable in the long term (Vlaicu et al. 2024).

## Final considerations

In western Pará, river transport of cattle is fundamental to Amazonian livestock systems, facilitating the movement of animals and the integration of the production chain. Therefore, this activity presents significant challenges related to animal welfare, along with adverse environmental factors, stocking density, long travel time and constant movement of vessels. Identifying and managing these factors is of great importance to ensure that the animals arrive at their destination in favorable conditions of comfort and health, preserving the quality of the carcass and the productivity of the sector. Implementation of good management practices, adequate infrastructure and constant monitoring strategies emerges as an effective way to alleviate stress and prevent injuries during transport. Aspects such as non-slip ramps and floors, adequate ventilation, water and food supply, along with trained workers, are crucial to promote animal welfare. The addition of these measures with technologies for monitoring and evaluating behavioral and physiological indices will support the safety and efficiency of river transport, making it ethical and sustainable.

## Data Availability

Not applicable.

## References

[CR1] Abdul-Majid M, Zahari SA, Othman N, Nadzri S (2024) Influence of technology adoption on farmers’ well-being: systematic literature review and bibliometric analysis. Heliyon 10(2):e24316. 10.1016/j.heliyon.2024.e24316 10.1016/j.heliyon.2024.e24316 PMC10835251 38312653

[CR100] Abubakar AA, Zulkifli I, Goh YM, Kaka U, Sabow AB, Imlan JC, Awad EA, Othman AH, Raghazli R, Mitin H, Sazili AQ (2021) Effects of housing and transport conditions on the physicochemical properties of meat and acute-phase proteins in cattle. Foods 10:252 10.3390/foods10020252 PMC7912028 33530479

[CR2] Acipar L, Queiroz KOD (2025) Vulnerability of the territory and regional circulation: the impacts of severe droughts in the municipality of Tefé in Amazonas. Soc Devel J 14(5). 10.33448/rsd-v14i5.48876

[CR3] Akhigbe BI, Munir K, Akinade O, Akanbi L, Oyedele LO (2021) IoT technologies for livestock management: a review of present status, opportunities, and future trends. Big Data Cognit Comput 5(1):10. 10.3390/bdcc5010010

[CR4] Andrade END, Roça RDO, Silva RAMS, Gonçalves HC, Pinheiro RSB (2008) Prevalence of lesions in carcasses of beef cattle slaughtered in the Pantanal of Mato Grosso do Sul transported by waterways. Braz J Anim Health Prod 9(4):789–795

[CR5] Bailone RL (2019) Export of live animals and animal welfare in Brazil: an overview of the current situation. J Educ Chang Continuing Educ Vet Med Anim Sci CRMV-SP 17(1):34–38. 10.36440/recmvz.v17i1.37841

[CR6] Bicalho AMDSM, Hoefle SW (2015) Conservation units, environmental services and frontier peasants in the Central Amazon: multi-functionality, Juxtaposition or conflict?. Res Econ Anthropol 35(1):65–105. 10.1108/S0190-128120150000035004

[CR9] Brazil (2013) Ministry of transport. In: Strategic Waterway Plan (PHE): plan report. Ministry of Transport; Arcadis Logos Consortium, Brasília

[CR7] Brazil (2019) Ministry of Agriculture, Livestock and Supply. In: Good practices in the export of live animals. MAPA, Brasília

[CR8] Brazil (2021) Ministry of Agriculture, Livestock and Supply. In: Vigiagro’s manual. MAPA, Brasília.

[CR10] Buckham-Sporer K, Earley B, Marti S (2023) Current knowledge on the transportation by road of cattle, including unweaned calves. Animals 13(21):3393. 10.3390/ani13213393 37958148 10.3390/ani13213393 PMC10649969

[CR11] Caitano TBSD, Homma AKO, Santos MASD, Brazil EC, Beltrão NES (2023) Technological profile of cattle ranching in Pará and the challenges of pasture sustainability. Colóquio - J Reg Devel 20(4):253–277

[CR12] Cislaghi TP, Brancher ME, Wegner D, Fernandes EB (2023) Live animal transportation and sustainable supply chain: a systematic literature review. Rev Adm UFSM 16(1):31. 10.5902/1983465973406

[CR13] Cortner O, Garrett RD, Valentim JF, Ferreira J, Niles MT, Reis J, Gil J (2019) Perceptions of integrated crop-livestock systems for sustainable intensification in the Brazilian Amazon. Land Use Policy 82:841–853. 10.1016/j.landusepol.2019.01.006

[CR14] Costa MJPD (2024) Handling and transport of cattle. Livest Handling Transp 6(10):213

[CR15] Damtew A, Erega Y, Ebrahim H, Tsegaye S, Msigie D (2018) The effect of long distance transportation stress on cattle: a review. Biomed J Sci Tech Res 3:3304–3308. 10.26717/BJSTR.2018.03.000908

[CR19] Džermeikaitė K, Bačėninaitė D, Antanaitis R (2023) Innovations in cattle farming: application of innovative technologies and sensors in the diagnosis of diseases. Animals 13(5):780. 10.3390/ani13050780 36899637 10.3390/ani13050780 PMC10000156

[CR16] Dias-Filho MB, Lopes SP (2020) History and challenges of cattle ranching in the Amazon. Embrapa. ISSN 1983-0513

[CR17] Dourado RR, Silva TF, Costa ML, Oliveira PA (2024) Port dynamics in the Amazon: changes in land use and land cover in the Área Verde neighborhood in Santarém (PA). Braz J Manag Reg Devel 20(2). ISSN 1809-239X

[CR18] Dunston-Clarke E, Willis RS, Fleming PA, Barnes AL, Miller DW, Collins T (2020) Developing an animal welfare assessment protocol for livestock transported by sea. Animals 10(4):705. 10.3390/ani10040705 32316532 10.3390/ani10040705 PMC7222738

[CR20] Emmanuel UO, Enekwenchi O, Dangrap NN, Oshibanjo OD (2025) Structural design and its effect on animal production: a review. Niger J Anim Production 1:1227–1230

[CR21] Espinoza JC, Marengo JA, Silva MS, Alves LM The new record of drought and warmth in the Amazon in 2023 related to regional and global climatic features. Sci Rep 14:2024. 10.1038/s41598-024-58782-5 10.1038/s41598-024-58782-5 PMC10998876 38582778

[CR22] Fernandes JN, Hemsworth PH, Coleman GJ, Tilbrook AJ (2021) Costs and benefits of improving farm animal welfare. Agriculture 11(2):104. 10.3390/agriculture11020104

[CR23] Fike K, Spire MF (2006) Transportation of cattle. Vet Clin N Am Food Anim Pract 22(2):305–320. 10.1016/j.cvfa.2006.03.012 10.1016/j.cvfa.2006.03.012 16814019

[CR24] Fleming PA, Wickham SL, Dunston-Clarke EJ, Willis RS, Barnes AL, Miller DW, Collins T (2020) Review of livestock welfare indicators relevant for the Australian live export industry. Animals 10(7):1236. 10.3390/ani10071236 32708293 10.3390/ani10071236 PMC7401645

[CR25] Garcia E, Ramos-Filho FSV, Mallmann GM, Fonseca F (2017) Costs, benefits and challenges of sustainable livestock intensification in a major deforestation frontier in the Brazilian Amazon. Sustainability 9(1):158. 10.3390/su9010158

[CR26] Gatti F, Higa TCCDS, Oyamada GC, Tamandaré JGS (2021) Advance of export cattle ranching in Pará: logistical aspects. Geogr Sci (Bauru) 25(1):325–356. Available at: https://www.agbbauru.org.br/publicacoes/revista/anoXXV_1/agb_xxv_1_web/agb_xxv_1-24.pdf

[CR27] Grandin T (2019a) Behavioral principles of beef cattle management and design of corrals, stables, races and loading ramps. In: Manejo e transporte de catado. CABI, Wallingford, United Kingdom, pp 80–109

[CR28] Grandin T (2019b) Livestock handling and transport, 5 edn. CABI, Wallingford, pp 496. 10.1079/9781786399151.0000

[CR29] Grandin T (2021) Cattle and pigs that are easy to move and handle will have less pre slaughter stress. Animals 10:2583 10.3390/foods10112583 PMC8625456 34828865

[CR30] Hansen BG, Langseth E, Berge C (2023) Animal welfare and cow-calf contact-farmers’ attitudes, experiences and adoption barriers. J Retailing Rural Stud 97:34–46. 10.1016/j.jrurstud.2022.11.013

[CR31] Heiderscheit KJ, Freestone AD, Beenken AM, Deters EL, Peschel JM, Hansen SL (2022) Long-duration transit and food and water deprivation alter behavioral activities and aggressive interactions at the feed bunk in beef feedlot steers. J Anim Sci 100(3):skac060. 10.1093/jas/skac060 10.1093/jas/skac060 PMC9030233 35226738

[CR32] Hing S, Foster S, Evans D (2021) Animal welfare risks in live cattle export from Australia to China by sea. Animals 11(10):2862. 10.3390/ani11102862 34679886 10.3390/ani11102862 PMC8532794

[CR33] Hogan JP, Petherick JC, Phillips CJ (2007) The physiological and metabolic impacts on sheep and cattle of feed and water deprivation before and during transport. Nutr Res Rev 20(1):17–28. 10.1017/S0954422407745006 19079858 10.1017/S0954422407745006

[CR34] Idris M, Uddin J, Sullivan M, Mcneill DM, Phillips CJC (2021) Non-invasive physiological indicators of heat stress in cattle. Animals 11(1):71. 10.3390/ani11010071 33401687 10.3390/ani11010071 PMC7824675

[CR35] Islam MA, Lomax S, Doughty A, Islam MR, Jay O, Thomson P, Clark C (2021) Automated monitoring of cattle heat stress and its mitigation. Front Anim Sci 2:737213. 10.3389/fanim.2021.737213

[CR36] Júnior APS, Lima NC, Júnior ACP, Oliveira JHCD (2011) A study of logistics for the flow of livestock production in the south of Pará 9(2):94–107

[CR37] Keeling LJ, Winckler C, Hintze S, Forkman B (2021) Towards a positive welfare protocol for cattle: a critical review of indicators and suggestion of how we might proceed. Front Anim Sci 2:753080. 10.3389/fanim.2021.753080

[CR38] Leão DSD, Leitão-Barboza MS, Prestes-Carneiro G (2023) Breeding and grazing of cattle (Bos taurus) in floodplain areas of the region of Santarém, Pará, an Amazonian context. Rev Arqueol 36(3):135–151

[CR39] Lilienthal G, Ahmad N, Mustafa F (2019) The live animal export trade. Eur Food Feed Law Rev 14(4):347–364

[CR40] Lima MA, Souza RT, Carvalho PR, Santos LA (2024) Severe droughts reduce river navigability and isolate communities in the Brazilian Amazon. Commun Earth Environ 5. 10.1038/s43247-024-01530-

[CR41] Ljungberg D, Gebresenbet G, Aradom S (2007) Logistics chain of animal transport and abattoir operations. Biosyst Eng 96(2):267–277. 10.1016/j.biosystemseng.2006.11.003

[CR42] Lourenço DB, Ludolf RVE (2020) The export of live cattle in Brazil and the constitutional rule of the prohibition of cruelty. Braz J Anim Law 15(3):53–73

[CR44] Ludolf RVE, Costa SRRD (2020b) The export of live cattle in Brazil and the constitutional rule of the prohibition of cruelty: a case study on the ship MVNADA. Confluences 22(1):101–119. Available at: https://periodicos.uff.br/confluencias/article/download/38245/23584/138703

[CR43] Ludolf RVE, Costa SRR da (2020a) The export of live cattle in Brazil and the constitutional rule of the prohibition of cruelty: a case study on the MV NADA ship. Confluences 22(1):101–119

[CR45] Ludolf RVE, Morgado EP, Oliveira FAGD, Chaves LA (2022) Maritime export of live cattle: legacies of colonial speciesism. Confluences 24(3):241–265. 10.22409/conflu.v24i3

[CR46] Martorano LG, Moraes JRDSCD, Silva LKX, Fernandes PCC, Amaral JMD, Lisboa LS, Neves KAL, Pacheco A, Beldini TP, Aparecido LEDO, Silva WCD, Godinho VDPC (2021) Agricultural and livestock production in the Amazon: a reflection on the necessity of adoption of integrated production strategies in the western region of the state of Pará. Aust J Crop Sci 15(8):1102–1109

[CR47] Masunga M, Mwendapole M (2025) Assessment of the impact of sea transportation on livestock welfare in Tanzania: a case study of dar es salaam port. IJSAT-Int J Sci Technol 16(4). 10.71097/IJSAT.v16.i4.8891

[CR48] Melasari T, Guntoro B, Muzayyanah A, Ilham N, Qui NH, Malindo R (2025) Implementation of animal welfare standards on livestock vessels and their impact on cattle weight loss and financial benefits. Anim Vet Sci 13(7):1586–1596. 10.17582/journal.aavs/2025/13.7.1586.1596

[CR49] Mishra S, Sharma SK (2023) Advanced contribution of IoT in agricultural production for the development of smart livestock environments. Internet Things 22:100724. 10.1016/j.iot.2023.100724

[CR50] Montalván S, Arcos P, Sarzosa P, Rocha RA, Yoo SG, Kim Y (2024) Technologies and solutions for cattle tracking: a review of the state of the art. Sensors 24(19):6486. 10.3390/s24196486 39409526 10.3390/s24196486 PMC11479337

[CR51] Neto GVFC (2021) Occurrence of contusions in bovine carcasses in the state of Pará due to transport 15(1):70–74. 10.26605/medvet-v15n1-2268

[CR52] Nielsen SS, Alvarez J, Bicout DJ, Calistri P, Canali E, Drewe JA, Garin-Bastuji B, Rojas JLG, Schmidt CG, Michel V, Chueca MÁM, Padalino B, Pasquali P, Roberts HC, Spoolder H, Stahl K, Velarde A, Viltrop A, Winckler C, Earley B, Edwards S, Faucitano L, Marti S, Lama GCMDL, Costa LN, Thomsen PT, Ashe S, Mur L, Stede YVD, Herskin M (2022) Welfare of cattle during transport. EFSA J 20(9):e07442. 10.2903/j.efsa.2022.7442 10.2903/j.efsa.2022.7442 PMC9449995 36092766

[CR53] North MA, Franke JA, Ouweneel B, Trisos CH (2023) Global risk of heat stress to cattle from climate change. Environ Res Lett 18(9):094027. 10.1088/1748-9326/aceb79

[CR101] Page MJ, McKenzie JE, Bossuyt PM, Boutron I, Hoffmann TC, Mulrow CD, Shamseer L, Tetzlaff JM, Akl EA, Brennan SE, Chou R (2021) The PRISMA 2020 statement: an updated guideline for reporting systematic reviews. BMJ 372:n71. 10.1136/bmj.n71 10.1136/bmj.n71 PMC8005924 33782057

[CR54] Papakonstantinou GI, Voulgarakis N, Terzidou G, Fotos L, Giamouri E, Papatsiros VG (2024) Precision livestock farming technology: applications and challenges of animal welfare and climate change. Agriculture 14(4):620. 10.3390/agriculture14040620

[CR55] Phillips C (2024) Transport of cattle, sheep and other livestock by sea and air. Livest Handling Transp 22(1):488–504. 10.1079/9781800625136.002

[CR56] Phillips CJ (2008) The welfare of livestock during sea transport. in: long distance transport and welfare of farm animals. Cabi, Wallingford, UK, pp 137–156

[CR57] Phillips CJC, Santurtun E (2013) The welfare of livestock transported by ship. Vet J 196(3):309–314. 10.1016/j.tvjl.2013.01.007 23473873 10.1016/j.tvjl.2013.01.007

[CR58] Ramos AK, Mcginley M, Carlo G (2021) The relations of workplace safety, perceived occupational stress, and adjustment among Latino/a immigrant cattle feedyard workers in the United States. Saf Sci 139:105262. 10.1016/j.ssci.2021.105262

[CR59] Rebezov M, Khayrullin M, Assenova B, Farida S, Baydan D, Garipova L, Savkina R, Rodionova S (2024) Improving meat quality and safety: innovative strategies. Slovak J Food Sci 18(1):523–546. 10.5219/1972

[CR60] Rebouças GF, Rosanova C (2021) Quantification of lesions and contusions in bovine carcasses resulting from pre-slaughter management and transport. In: Animal science: applied animal science, 1 edn, vol 1. Publisher Conhecimento Livre, pp 7–15. 10.37423/201203432

[CR61] Reis GG, Molento CFM (2020) Emerging market multinationals and international corporate social responsibility standards: bringing animals to the fore. J Bus Ethics 166(2):351–368. 10.1007/s10551-019-04144-5

[CR62] Reyes F (2024) Dairy cattle handling and transport. Livest Handling Transp 6:122–137. 10.1079/9781800625136.0006

[CR63] Rodrigues-Junior UJ, Dziedzic M (2021) The water footprint of beef cattle in the amazon region, Brazil. Ciência Rural 51:20190294. 10.1590/0103-8478cr20190294

[CR64] Rohleder LAS, Querino CAS, Alves PV et al (2022) Evaluation of environmental parameters in a microregion in southern Amazonas state, Brazil, and their relationship with heat stress in dairy cattle. Ciênc Anim Bras 23:e71625E. 10.1590/1809-6891v23e-71625E

[CR65] Santos TFC, Santos IFCD, Cruz CFD, Rosa VBB, Limberger RA, Vitorio HS, Ferreira E, Pazdiora RD, Andrade ER (2025) Impact of dry and rainy seasons on physiological and behavior aspects of crossbreed dairy cows of amazon biome of Costa Marques, Rondônia, Brazil. Cad Pedag 22(5):e15224–e15224. 10.54033/cadpedv22n5-292

[CR66] Sarubbi J, Martínez-Burnes J, Ghezzi MD, Olmos-Hernandez A, Lendez PA, Ceriani MC, Hernández-Avalos I (2024) Hypothalamic neuromodulation and control of the dermal surface temperature of livestock during hyperthermia. Animals 14(12):1745. 10.3390/ani14121745 38929364 10.3390/ani14121745 PMC11200636

[CR67] Schuetze SJ, Schwandt EF, Maghirang RG, Thomson DU (2017) Transportation of commercial finished cattle and animal welfare considerations. Prof Anim Sci 33(5):509–519. 10.15232/pas.2017-01620

[CR68] Shephard RW, Maloney SK (2023) A review of thermal stress in cattle. Aust Vet J 101(11):417–429. 10.1111/avj.13275 37620993 10.1111/avj.13275

[CR69] Shin H, Kwak Y, Jo SK, Kim SH, Huh JH (2022) Applicability evaluation of a demand-controlled ventilation system in livestock. Comput Electron Agric 196:106907. 10.1016/j.compag.2022.106907 35368438 10.1016/j.compag.2022.106907 PMC8963795

[CR71] Silva TM, Brainer MMDA, Godoy HBRD, Paiva SC, Neto RF (2022b) Influence of transport on the welfare and quality of beef meat. Open Sci Res VI C 144:2094–2112. 10.37885/220910116

[CR70] Silva TM, Brainer MMDA, Godoy HBR, Paiva SC, Neto RF (2022a) Influence of transport on the welfare and quality of beef meat. Digit Sci Publishing House 6:2094–2112. ISBN: 978-65-5360-212-0. 10.37885/220910116

[CR72] Silva WCD, Fontenele LV, Neto OGN, Silva ÉBRD, Camargo-Júnior RNC, Sousa CEL, Belo TS, Silva JARD, Lourenço-Júnior JDB (2024a) Beef consumers’ perception of welfare on animal farm slaughter in Altamira, Pará, Brazil. Rev Salud Anim 46(17):1–7. https://cu-id.com/2248/v46e17

[CR73] Silva WCD, Gouveia Júnior A, Damasceno NMS, Sousa LF, Barbosa AVC, Silva ÉBR, Silva AGME (2024b) Effect of transportation distance and lairage time on selected behaviors and carcass parameters in zebu cattle-a study using the animal focal sampling method. Front Vet Sci 11:1385481. 10.3389/fvets.2024.1385481 38840627 10.3389/fvets.2024.1385481 PMC11150803

[CR74] Silva WCD, Silva JAR, Martorano LG, Silva ÉBR et al (2024c) Thermal comfort of Nelore cattle (Bos indicus) managed in silvopastoral and traditional systems associated with rumination in a humid tropical environment in the Eastern Amazon, Brazil. Vet Sci 11(6):236. 10.3390/vetsci11060236 38921983 10.3390/vetsci11060236 PMC11209581

[CR75] Silva WCD, Silva JAR, Silva ÉBR, Barbosa AVC, Sousa CEL et al (2023) Characterization of thermal patterns using infrared thermography and thermolytic responses of cattle reared in three different systems during the transition period in the Eastern Amazon, Brazil. Animals 13(17):art. 2735. 10.3390/ani13172735 10.3390/ani13172735 PMC10487038 37685000

[CR76] Sims JT, Bergström L, Bowman BT, Oenema OJSU (2005) Nutrient management for intensive animal agriculture: policies and practices for sustainability. Soil Use Manag 21:141–151. 10.1111/j.1475-2743.2005.tb00418.x

[CR77] Singh C, Goswami M, Pathak V (2024) Pre-harvest management of meat animals and poultry: care and transportation. J Anim Feed Sci Technol 12(2):6. 10.21088/jafst.2321.1628.12224.2

[CR78] Souza Galvão DE, J J, Teixeira MM (2025) Livestock and animal welfare: from property to slaughter. Ibero-Am J Humanit Sci Educ 11(6):1873–1885. 10.51891/rease.v11i6.19819

[CR79] Souza IMFD, Sousa CEL, Pinto VS, Vilela LGP, Silva Souza AD, Sousa Cunha JPD, Da Silva WC (2026) Welfare indicators in cattle farming in the face of heat stress: a review in climate change scenarios. Front Vet Sci 12:1754412 41756016 10.3389/fvets.2025.1754412 PMC12932167

[CR80] Sristi PR, Das NR, Akhter A, Kaniya NM, Hashem MA (2025) Relation among meat pH, color and tenderness: a review. Meat Res 5(3):1–7. 10.55002/mr.5.3.117

[CR81] Sullivan PA, Davis M, Nair MN, Hess AM, Mooney DF, Edwards-Callaway LN (2024) Preslaughter factors affecting mobility, blood parameters, bruising, and muscle pH of finished beef cattle in the United States. Transl Anim Sci 8. 10.1093/tas/txae035 10.1093/tas/txae035 PMC10983080 38562213

[CR82] Tavares NJB, Neto AP (2025) Logistical challenges of Amazonian river transport: evaluation of the difficulties faced in the movement of goods in the Amazon river in the dry period of the rivers. J Appl Soc Sci 29(144). 10.69849/revistaft/ra10202503111820

[CR83] Toledo IM, Dahl GE, Vries AD (2022) Dairy cattle management and housing for warm environments. Livest Sci 255:104802. 10.1016/j.livsci.2021.104802

[CR84] Uchendu C, Ayo JO, Tekdek LB, Zakari FO (2022) Impact of road transportation on physiological and oxidative stress biomarkers in puppies. J Therm Biol 110:103376. 10.1016/j.jtherbio.2022.103376 36462888 10.1016/j.jtherbio.2022.103376

[CR85] Valadez NM, Estévez MLX, Galindo F, Pérez MF, Villarroel M, Miranda-De La Lama GC (2022) Consequences of long-distance transport on the behavior and health of young-bulls that may affect their fitness to adapt to feedlots. Livest Sci 265:105083. 10.1016/j.livsci.2022.105083

[CR87] Vlaicu PA, Gras MA, Untea AE, Lefter NA, Rotar MC (2024) Advancing livestock technology: intelligent systemization for enhanced productivity, welfare, and sustainability. AgriEngineering 6(2):1479–1496. 10.3390/agriengineering6020084

[CR86] Válková L, Večerek V, Voslářová E, Kaluža M, Takáčová D (2021) The welfare of cattle, sheep, goats and pigs from the perspective of traumatic injuries detected at slaughterhouse postmortem inspection. Animals 11(5):1406. 10.3390/ani11051406 34069150 10.3390/ani11051406 PMC8156928

[CR88] Wilmann M, Hoffmann G, Genísio AO, Tonon FC, Lancet AD, Corrêa AHP, Peetz LR (2020) Effect of rest stop duration during long-distance transport on welfare indicators of cattle. PLoS ONE 15(4):e0228492. 10.1371/journal.pone.0228492

[CR89] World Organisation for Animal Health (WOAH) (2021) Terrestrial animal health code. Chapter 7.3 - transport of animals by land and sea. WOAH, Paris

[CR90] Zanardi E, Luca SD, Alborali GL, Ianieri A, Varrà MO, Romeo C, Ghidini S (2022) Relationship between bruises on carcasses of beef cattle and transport-related factors. Animals 12(15):1997. 10.3390/ani12151997 35953986 10.3390/ani12151997 PMC9367580

